# Candidate drugs for preventive treatment of unruptured intracranial aneurysms: A cross-sectional study

**DOI:** 10.1371/journal.pone.0246865

**Published:** 2021-02-12

**Authors:** Kampei Shimizu, Hirotoshi Imamura, Shoichi Tani, Hidemitsu Adachi, Chiaki Sakai, Akira Ishii, Hiroharu Kataoka, Susumu Miyamoto, Tomohiro Aoki, Nobuyuki Sakai

**Affiliations:** 1 Department of Neurosurgery, Kobe City Medical Center General Hospital, Kobe, Hyogo, Japan; 2 Department of Neurosurgery, Kyoto University Graduate School of Medicine, Kyoto, Japan; 3 Department of Molecular Pharmacology, Research Institute, National Cerebral and Cardiovascular Center, Osaka, Japan; Barrow Neurological Institute, UNITED STATES

## Abstract

**Background and purpose:**

Establishment of drug therapy to prevent rupture of unruptured intracranial aneurysms (IAs) is needed. Previous human and animal studies have gradually clarified candidate drugs for preventive treatment of IA rupture. However, because most of these candidates belong to classes of drugs frequently co-administered to prevent cardiovascular diseases, epidemiological studies evaluating these drugs simultaneously should be performed. Furthermore, because drugs included in the same class may have different effects in terms of disease prevention, drug-by-drug assessments are important for planning intervention trials.

**Materials and methods:**

We performed a cross-sectional study enrolling patients diagnosed with IAs between July 2011 and June 2019 at our institution. Patients were divided into ruptured or unruptured groups. The drugs investigated were selected according to evidence suggested by either human or animal studies. Univariate and multivariate logistic regression analyses were performed to assess the association of drug treatment with rupture status. We also performed drug-by-drug assessments of the association, including dose-response relationships, with rupture status.

**Results:**

In total, 310 patients with ruptured and 887 patients with unruptured IAs were included. Multivariate analysis revealed an inverse association of statins (odds ratio (OR), 0.54; 95% confidence interval (CI) 0.38–0.77), calcium channel blockers (OR, 0.41; 95% CI 0.30–0.58), and angiotensin II receptor blockers (ARBs) (OR, 0.67; 95% CI 0.48–0.93) with ruptured IAs. Moreover, inverse dose-response relationships with rupture status were observed for pitavastatin and rosuvastatin among statins, benidipine, cilnidipine, and amlodipine among calcium channel blockers, and valsartan, azilsartan, candesartan, and olmesartan among ARBs. Only non-aspirin non-steroidal anti-inflammatory drugs were positively associated with ruptured IAs (OR, 3.24; 95% CI 1.71–6.13).

**Conclusions:**

The present analysis suggests that several types of statins, calcium channel blockers, and ARBs are candidate drugs for preventive treatment of unruptured IAs.

## Introduction

Preventive treatment by neurosurgical or endovascular intervention has been applied for patients with unruptured intracranial aneurysms (IAs) when the estimated rupture rate outweighed the risk of complications associated with these modalities; otherwise, patients were untreated and observed by serial imaging [1]. Currently, the establishment of drug therapy to prevent the rupture of IAs is a central subject in this field.

Past observational studies comparing ruptured and unruptured IAs have implied that several drugs, such as statins or aspirin, decrease the risk of rupture [2, 3]. More recent studies have reproduced these findings with a higher volume of cases and using multivariate analyses with propensity score weighting [4, 5]. Because these results are consistent with the evidence acquired from animal studies [6, 7], the development of drug therapy for unruptured IAs has become more feasible.

Several issues have, however, not been elucidated. For instance, because most of the drugs proposed thus far are typically used to prevent cardiovascular diseases, these drugs are frequently co-administered in a single patient [2–5, 8]. Observational studies evaluating these drugs simultaneously should thus be useful to assess the association with rupture status more precisely. Furthermore, even drugs included in the same class may have different effects in terms of disease prevention [9]. Thus, drug-by-drug assessments are useful.

We, therefore, performed a cross-sectional study using a database from a high-volume center to identify drugs associated with the rupture status of IAs. The drugs evaluated in the present study were selected by referencing evidence provided by either human [2–5, 8] or animal studies [10–12]. Furthermore, drugs belonging to classes that were of interest according to a multivariate analysis were evaluated regarding the association of each drug with rupture status and the dose-response relationship. The results obtained in the present study will provide further insight into the development of preventive drug treatment for unruptured IAs.

## Materials and methods

The institutional review board at Kobe City Medical Center General Hospital approved the study protocol. Informed consent from individual patients was waived by the board because of the minimal risk associated with the present study. Alternatively, an opt-out enrolment of patients was applied. The study followed the Strengthening the Reporting of Observational Studies in Epidemiology (STROBE) statement [13]. The datasets analyzed in the present study are provided in S1 Table (i.e., main dataset, n = 1197) and S2 Table (i.e., subgroup dataset, n = 920).

### Study population

Patients who received medical care at our institution between July 2011 and June 2019 were assessed for eligibility. The inclusion criteria consisted of patients with (1) ruptured IAs or (2) unruptured IAs ≥ 3 mm in the largest dimension. The exclusion criteria were as follows: (1) infectious aneurysm, (2) dissecting aneurysm, or (3) internal carotid artery aneurysm located proximal to the posterior communicating artery (i.e., paraclinoid aneurysms). Paraclinoid aneurysms were excluded because of their lower annual rupture rate and larger female predominance compared with aneurysms at other locations [14, 15].

The patient and aneurysmal characteristics analyzed in the present study included age, sex, smoking habits, medical history of aneurysmal subarachnoid hemorrhage (aSAH), familial history of aSAH, and size of aneurysms.

### Collection of drug therapy data

The drugs investigated in the present study were selected based on evidence from previous human [2–5, 8] or animal studies [10–12]. Drugs included calcium (Ca) channel blockers, angiotensin II receptor blockers (ARBs), angiotensin-converting enzyme inhibitors, renin inhibitors, thiazide/indapamide, β-adrenergic receptor blockers, 3-hydroxy-3-methylglutaryl coenzyme A (HMG-CoA) reductase inhibitors (statins), eicosapentaenoic acid, dipeptidyl peptidase-4 inhibitors, peroxisome proliferator-activated receptor γ agonists, biguanides, selective serotonin reuptake inhibitors, glucocorticoids, anticoagulants, non-aspirin non-steroidal anti-inflammatory drugs (non-aspirin NSAIDs), selective cyclooxygenase (COX)-2 inhibitors, and selective estrogen receptor modulators. Aspirin and other anti-platelet agents (i.e., clopidogrel, cilostazol, prasugrel, or dipyridamole) were included in the sensitivity analysis as described in the following section. Drug users were defined as patients who took each drug at the time of diagnosis on a daily basis.

### Statistical analysis

Differences between ruptured and unruptured IAs were assessed as follows: continuous variables were evaluated with Student’s t-test, and categorical variables were assessed with the Pearson χ^2^ test or Fisher exact test as appropriate. Regarding the association between drug treatment and rupture status, drugs were first categorized into classes based on the mechanism of action (e.g., Ca channel blockers). Univariate and multivariate logistic regression models were used to assess the association of these classes of drugs with rupture status. A *P*-value less than 0.20 in the univariate analysis was applied as the cut-off value for the multivariate logistic regression model. For classes that were significantly associated with rupture status in the multivariate logistic regression model, drug-by-drug assessments were further performed to evaluate differences within each class. When assessing dose-response relationships with rupture status, the doses were divided into two groups. If more than two doses were used for a single drug, the doses were divided into two groups so that the numbers of each dose group were as even as possible. The associations were assessed by calculating the proportion (%) and odds ratio (OR) with the 95% confidence interval (CI). Dose-response relationships were assessed with the Cochran-Armitage trend test. A *P* value less than 0.05 was defined as statistically significant.

**Fig 1:**
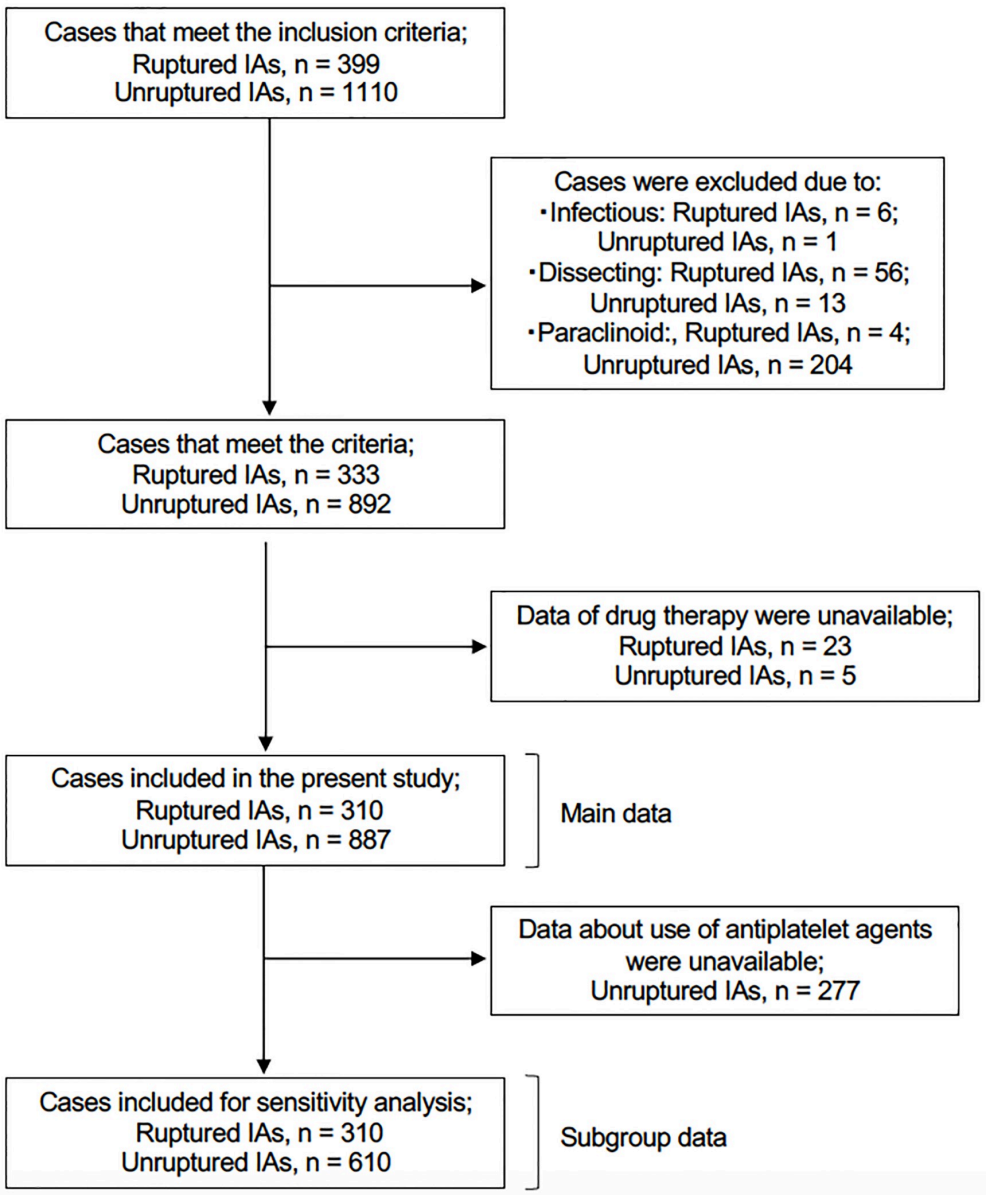
Case inclusion flowchart. IAs: intracranial aneurysms.

A sensitivity analysis using a subset of data was performed as follows. We initially planned to include anti-platelet drugs in the present analyses, considering the controversy associated with the use of aspirin as a potential treatment for unruptured IAs [16]. However, among patients with unruptured IAs treated endovascularly, we could not determine from our database whether these drugs were administered as a pretreatment for the procedures or at the time of diagnosis. Therefore, this class of drugs was not included in the main dataset. Instead, we created a subgroup for sensitivity analysis by excluding patients with unruptured IAs treated endovascularly (Fig 1). Data regarding the use of aspirin and other anti-platelet agents in this subgroup were included in the sensitivity analysis. All statistical analyses were performed with JMP software (version 14.0, SAS Institute, Cary, NC, USA).

## Results

### Association of each class of drugs with rupture status

A total of 333 patients with ruptured and 892 patients with unruptured IAs met the inclusion criteria. All of the patients were Japanese. Among these patients, drug treatment data were unavailable for 23 (6.9%) and 5 (0.6%) patients with ruptured and unruptured IAs, respectively. Data from 310 patients with ruptured and 887 patients with unruptured IAs were thus analyzed in the present study (Fig 1).

The baseline characteristics and drug treatments of enrolled patients are shown in Table 1. The univariate analyses revealed potential associations of Ca channel blockers, ARBs, statins, dipeptidyl peptidase-4 inhibitors, glucocorticoids, and non-aspirin NSAIDs with rupture status. The proportion of patients who were not taking any of these medications was significantly higher in the ruptured IA group (170/310, 54.8%) than in the unruptured IA group (273/887, 30.8%) (*P* < 0.0001). After adjustment of the logistic regression model, Ca channel blockers (OR, 0.41; 95% CI 0.30–0.58), ARBs (OR, 0.67; 95% CI 0.48–0.93), and statins (OR, 0.54; 95% CI 0.38–0.77) were inversely associated with ruptured IAs. Only non-aspirin NSAIDs were positively associated with ruptured IAs (OR, 3.24; 95% CI 1.71–6.13).

**Table 1:** Baseline characteristics and drug treatments of 310 patients with ruptured intracranial aneurysms and 887 patients with unruptured intracranial aneurysms.

Characteristics	Ruptured IA (n = 310)*	Unruptured IA (n = 887)*	Univariate analysis			Multivariate analysis		
			OR	95% CI	*P* value	OR	95% CI	*P* value
Age, y (SD)	63.2 (16.0)	62.8 (10.6)	1.00	0.99–1.01	0.58			
Women, n (%)	219 (70.7)	577 (65.1)	1.29	0.98–1.71	0.07	1.33	0.99–1.79	0.06
Smokers, n (%)	139 (44.8)	426 (48.0)	0.88	0.68–1.14	0.33			
History of aSAH, n (%)	11 (3.6)	51 (5.8)	0.60	0.31–1.17	0.13	0.53	0.26–1.09	0.09
Familial history of aSAH, n (%)	32 (10.3)	169 (19.1)	0.49	0.33–0.73	0.0004	0.5	0.33–0.76	0.001
Size, mm (SD)	7.0 (3.9)	6.5 (4.3)	1.02	0.99–1.05	0.10	1.03	1.00–1.06	0.049
Calcium channel blockers	63 (20.3)	368 (41.5)	0.36	0.26–0.49	< 0.0001	0.41	0.30–0.58	< 0.0001
Angiotensin II receptor blockers	73 (23.6)	336 (37.9)	0.51	0.38–0.68	< 0.0001	0.67	0.48–0.93	0.02
Angiotensin-converting enzyme inhibitors	8 (2.6)	27 (3.0)	0.84	0.38–1.88	0.68	-	-	-
Renin inhibitors	1 (0.3)	3 (0.3)	0.95	0.01–9.20	1.00	-	-	-
Thiazide/Indapamide	12 (3.9)	51 (5.8)	0.66	0.35–1.26	0.20	-	-	-
β-adrenergic receptor blockers	24 (7.7)	78 (8.8)	0.87	0.54–1.40	0.57	-	-	-
HMG-CoA reductase inhibitors (statins)	50 (16.1)	268 (30.2)	0.44	0.32–0.62	< 0.0001	0.54	0.38–0.77	0.0008
Eicosapentaenoic acid	8 (2.6)	28 (3.2)	0.81	0.37–1.80	0.61	-	-	-
Dipeptidyl peptidase-4 inhibitors	9 (2.9)	42 (4.7)	0.60	0.29–1.25	0.17	0.99	0.46–2.16	0.99
Peroxisome proliferator-activated receptor γ agonists	2 (0.7)	11 (1.2)	0.52	0.11–2.35	0.53	-	-	-
Biguanides	4 (1.3)	17 (1.9)	0.67	0.22–2.00	0.47	-	-	-
Selective serotonin reuptake inhibitors	5 (1.6)	15 (1.7)	0.95	0.34–2.64	0.93	-	-	-
Glucocorticoids	10 (3.2)	17 (1.9)	1.70	0.77–3.77	0.18	1.75	0.73–4.21	0.21
Non-aspirin non-steroidal anti-inflammatory drugs	24 (7.7)	23 (2.6)	3.15	1.75–5.67	< 0.0001	3.24	1.71–6.13	0.0003
Selective COX-2 inhibitors	8 (2.6)	15 (1.7)	1.54	0.65–3.67	0.33	-	-	-
Selective estrogen receptor modulators	1 (0.3)	11 (1.2)	0.26	0.03–2.00	0.32	-	-	-
Anticoagulants	6 (1.9)	20 (2.3)	0.86	0.34–2.15	0.74	-	-	-

*Data are shown as the n (%) or mean (standard deviation).

Abbreviations; CI = confidence interval, IA = intracranial aneurysm, OR = odds ratio, SD = standard deviation, aSAH = aneurysmal subarachnoid hemorrhage, HMG-CoA = 3-hydroxy-3-methylglutaryl coenzyme A, COX = cyclooxygenase.

Multivariate analysis using the subgroup data reproduced the association with rupture status for Ca channel blockers (OR, 0.41; 95% CI 0.29–0.60), ARBs (OR, 0.66; 95% CI 0.46–0.95), statins (OR, 0.61; 95% CI 0.41–0.90), and non-aspirin NSAIDs (OR, 2.48; 95% CI 1.26–4.90) (Table 2).

**Table 2:** Sensitivity analysis of data from the subgroup of patients.

Characteristics	Ruptured IA (n = 310)*	Unruptured IA (n = 610)*	Univariate analysis			Multivariate analysis		
			OR	95% CI	*P* value	OR	95% CI	*P* value
Age, y (SD)	63.2 (16.0)	62.5 (10.2)	1.00	0.99–1.02	0.44			
Women, n (%)	219 (70.7)	410 (67.2)	1.17	0.87–1.58	0.29			
Smokers, n (%)	139 (44.8)	288 (47.2)	0.91	0.69–1.20	0.49			
History of aSAH, n (%)	11 (3.6)	35 (5.7)	0.60	0.30–1.21	0.15	0.65	0.27–0.30	0.27
Familial history of aSAH, n (%)	39 (12.6)	157 (25.7)	0.42	0.28–0.61	< 0.0001	0.44	0.29–0.66	< 0.0001
Size, mm (SD)	7.0 (3.9)	5.5 (2.1)	1.19	1.13–1.26	< 0.0001	1.22	1.15–1.29	< 0.0001
Calcium channel blockers	63 (20.3)	235 (38.5)	0.41	0.30–0.56	< 0.0001	0.41	0.29–0.60	< 0.0001
Angiotensin II receptor blockers	73 (23.6)	225 (36.9)	0.53	0.39–0.72	< 0.0001	0.66	0.46–0.95	0.02
Angiotensin-converting enzyme inhibitors	8 (2.6)	23 (3.8)	0.68	0.30–1.53	0.34	-	-	-
Renin inhibitors	1 (0.3)	1 (0.2)	1.97	0.12–31.6	1.00	-	-	-
Thiazide/Indapamide	12 (3.9)	38 (6.2)	0.61	0.31–1.18	0.14	0.84	0.40–1.79	0.66
β-adrenergic receptor blockers	24 (7.7)	51 (8.4)	0.92	0.55–1.53	0.75	-	-	-
HMG-CoA reductase inhibitors (statins)	50 (16.1)	177 (29.0)	0.47	0.33–0.67	< 0.0001	0.61	0.41–0.90	0.01
Eicosapentaenoic acid	8 (2.6)	18 (3.0)	0.87	0.37–2.03	0.75	-	-	-
Dipeptidyl peptidase-4 inhibitors	9 (2.9)	29 (4.8)	0.6	0.28–1.28	0.18	0.86	0.38–1.95	0.71
Peroxisome proliferator-activated receptor γ agonists	2 (0.7)	6 (1.0)	0.65	0.13–3.26	0.72	-	-	-
Biguanides	4 (1.3)	15 (2.5)	0.52	0.17–1.58	0.24	-	-	-
Selective serotonin reuptake inhibitors	5 (1.6)	8 (1.3)	1.23	0.40–3.80	0.77	-	-	-
Glucocorticoids	10 (3.2)	9 (1.5)	2.23	0.89–5.54	0.08	2.51	0.87–7.28	0.09
Non-aspirin non-steroidal anti-inflammatory drugs	24 (7.7)	20 (3.3)	2.48	1.35–4.56	0.003	2.48	1.26–4.90	0.009
Selective COX-2 inhibitors	8 (2.6)	11 (1.8)	1.44	0.57–3.62	0.43	-	-	-
Selective estrogen receptor modulators	1 (0.3)	9 (1.5)	0.22	0.03–1.71	0.18	0.14	0.02–1.24	0.08
Anticoagulants	6 (1.9)	11 (1.8)	1.07	0.39–2.93	0.89	-	-	-
Aspirin	17 (5.5)	59 (9.7)	0.54	0.31–0.95	0.03	0.78	0.42–1.47	0.45
Anti-platelet agents	13 (4.2)	27 (4.4)	0.95	0.48–1.86	0.87	-	-	-

*Data are shown as the n (%) and mean (SD).

Abbreviations; CI = confidence interval, IA = intracranial aneurysm, OR = odds ratio, SD = standard deviation, aSAH = aneurysmal subarachnoid hemorrhage, HMG-CoA = 3-hydroxy-3-methylglutaryl coenzyme A, COX = cyclooxygenase.

Aspirin and other anti-platelet agents are additionally included in the sensitivity analysis.

### Association of individual drugs with rupture status

**Fig 2:**
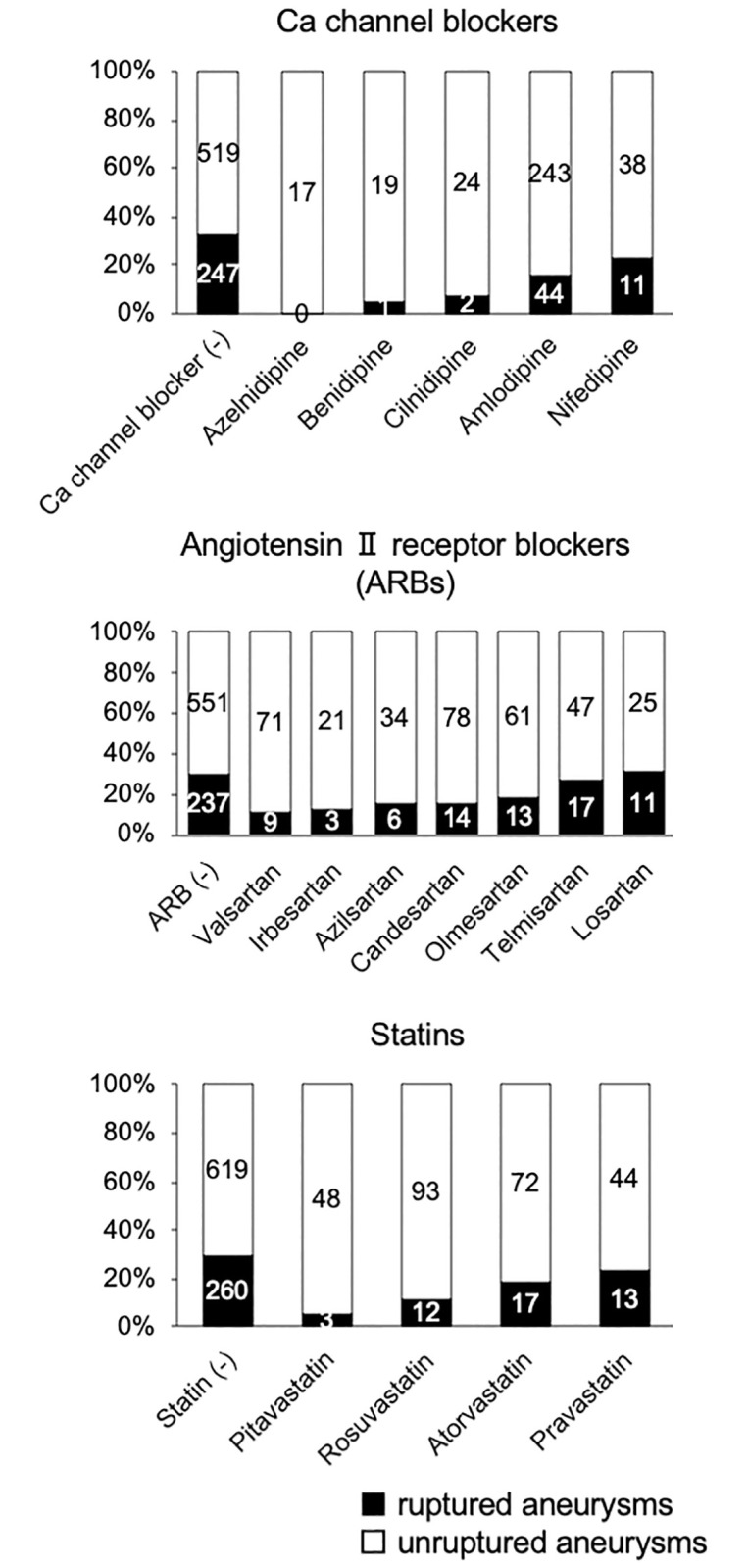
The proportion of ruptured intracranial aneurysms stratified by drug in the following classes: Calcium channel blockers, angiotensin II receptor blockers, and statins. The numbers of patients taking each drug are presented in the bar graphs.

According to the results of the multivariate analysis, the associations of each drug included in the Ca channel blocker, ARB, and statin classes with rupture status were further investigated. The drug-by-drug assessment revealed a difference in the association with rupture status among drugs within the same classes (Table 3). The use of nifedipine among Ca channel blockers, irbesartan, telmisartan, and losartan among ARBs, and pravastatin among statins was not significantly associated with rupture status (Fig 2 and Table 3). The numbers of patients taking each drug are provided in the bar graphs of Fig 2.

**Table 3:** Associations of individual drugs with ruptured intracranial aneurysms.

Drug	OR	95% CI	*P* value
Calcium channel blockers			
Azelnidipine	0	-	0.005
Benidipine	0.11	0.015–0.83	0.01
Cilnidipine	0.18	0.041–0.75	0.008
Amlodipine	0.38	0.27–0.54	< 0.0001
Nifedipine	0.61	0.31–1.21	0.15
Angiotensin II receptor blockers			
Valsartan	0.29	0.14–0.60	0.0004
Irbesartan	0.33	0.098–1.12	0.06
Azilsartan	0.41	0.17–0.99	0.04
Candesartan	0.42	0.23–0.75	0.003
Olmesartan	0.50	0.27–0.92	0.02
Telmisartan	0.84	0.47–1.49	0.55
Losartan	1.02	0.50–2.11	0.95
Statins			
Pitavastatin	0.15	0.046–0.48	0.0003
Rosuvastatin	0.31	0.17–0.57	< 0.0001
Atorvastatin	0.56	0.32–0.97	0.04
Pravastatin	0.7	0.37–1.33	0.28

Abbreviations: CI = confidence interval, OR = odds ratio.

### Association of dose with rupture status

**Fig 3:**
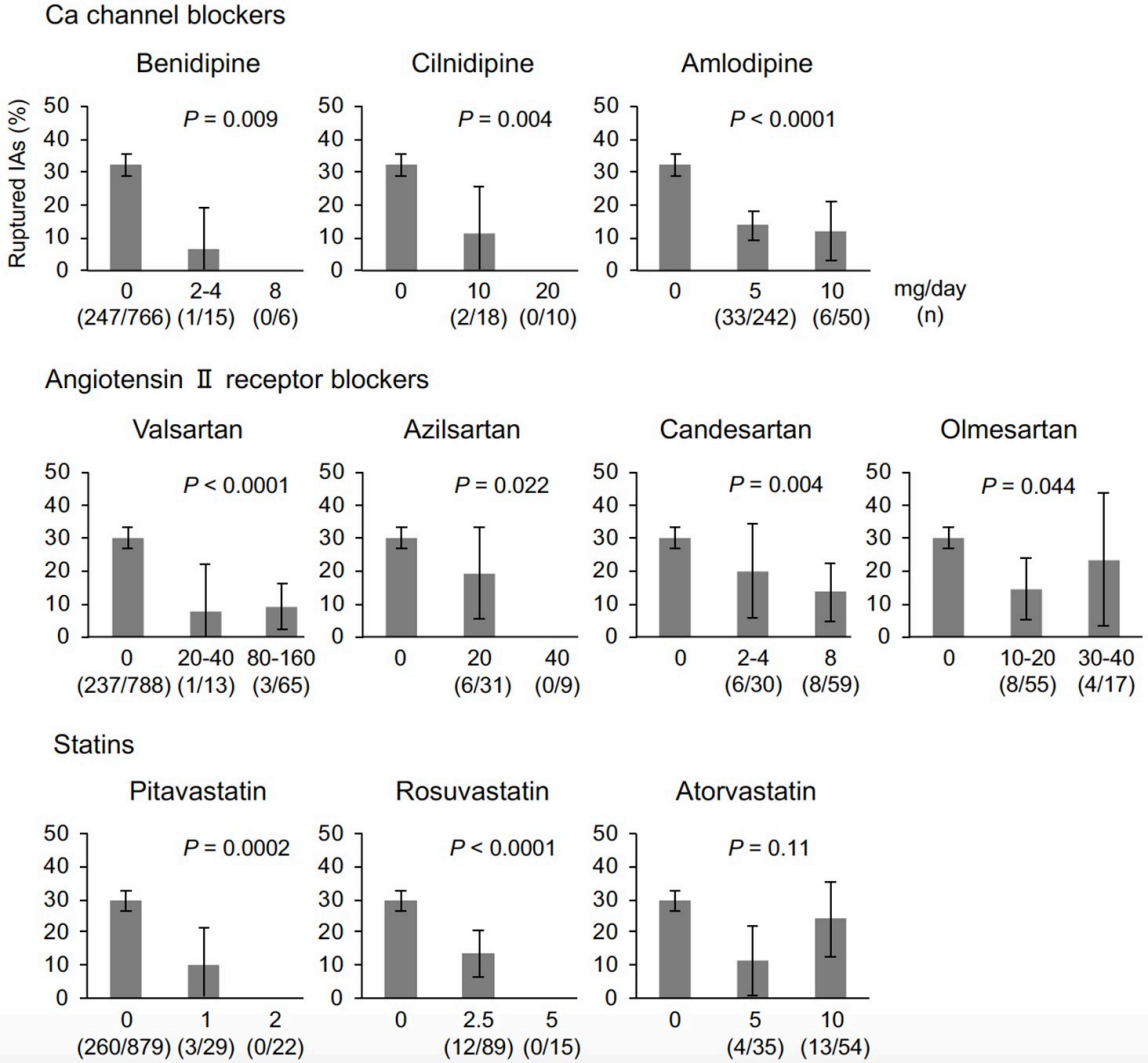
The proportion of ruptured intracranial aneurysms (IAs) stratified by drug and the dose per day. Data are presented as percentages and 95% confidence intervals.

Dose data were unavailable for amlodipine, valsartan, olmesartan, pitavastatin, and rosuvastatin in six, two, one, one, and three cases, respectively. A dose-response relationship with rupture status was not identified for atorvastatin (Fig 3). Inverse dose-response relationships with ruptured IAs were observed for the other drugs (Fig 3).

## Discussion

The present study suggested that Ca channel blockers, ARBs, and statins are reliable candidate drugs to prevent IA rupture. Interestingly, the degrees of the associations with rupture status were different among drugs, even those within the same class, indicating a difference in the efficacy of preventing IA rupture. Among Ca channel blockers, nifedipine was not associated with rupture status, which may be attributable to the differences in blocking potency for each subtype of Ca channel among this class of drugs; nifedipine mostly exerts its action on L-type Ca channels, while other Ca channel blockers also affect N-type and P/Q type Ca channels [17]. Statins more aggressively decrease low-density lipoprotein cholesterol, and so-called ‘strong statins’ (i.e., pitavastatin, rosuvastatin, and atorvastatin) [18] demonstrated a significant association with rupture status in the present analyses. According to the dose-response analysis, pitavastatin and rosuvastatin may be leading candidate drugs among statins to prevent aSAH. The results derived from the drug-by-drug analyses are especially important for planning intervention trials.

Because management of unruptured IAs consists of a preventive strategy, the development of less invasive therapy, such as drug therapy, is necessary. In a cross-sectional study enrolling 117 patients with ruptured and 304 patients with unruptured IAs, statin use was inversely associated with ruptured IAs with an OR of 0.30 (95% CI, 0.14–0.66) [2]. A recently published study reproduced these results by enrolling thousands of patients and adjusting baseline characteristics by propensity score weighting (OR, 0.41; 95% CI, 0.23–0.73) [5]. In the present study, the association of statins with ruptured IAs was reproduced even after adjusting for confounding by co-administered drugs such as Ca channel blockers and ARBs. Statins, therefore, may be leading candidates for therapeutic drugs to prevent aSAH.

In the present analyses, non-aspirin NSAIDs were positively associated with aSAH. This result may be attributable to an adverse effect of non-selective COX inhibition, i.e., suppressing the physiological production of prostaglandins, resulting in anti-platelet action or dysfunction of arterial autoregulation [19]. In fact, this result is conflicting among previous studies. In a meta-analysis, the association of non-aspirin NSAIDs with hemorrhagic stroke was not significant [20]. The discrepancy among related studies, including the present study, may be partly explained by racial differences among participants. Indeed, a meta-analysis demonstrated that intracranial hemorrhages associated with a non-selective COX inhibitor, aspirin, are more frequent in the Asian population [21]. The interpretation of the association of non-selective COX inhibitors with aSAH is especially important because aspirin has recently been recognized as a leading candidate drug for preventive treatment of unruptured IAs. A previous study demonstrated an inverse association of aspirin use with ruptured IAs, and the association depended on the frequency of aspirin use [3]. The potential of aspirin as a preventive drug therapy for unruptured IAs has been repeatedly reproduced in other studies [5, 22]. In this regard, the present sensitivity analysis data were not in conflict with those of past studies but should be regarded as inconclusive because of the small sample size and systematically missing data (Fig 1 and Table 2). However, another study showed a positive association of aspirin use with ruptured IAs [23]. A randomized intervention trial that is being conducted in Western countries will provide an answer to this controversy [24].

There are several limitations of the present study. First, this study is a retrospective cross-sectional study based on a single-center database. The duration of drug treatment before the diagnosis of IA and drug adherence were not determined. Furthermore, the enrolled patients consisted of a homogeneous population (i.e., Japanese patients). Second, selection bias attributable to the asymptomatic nature of unruptured IAs should be recognized. Patients with unruptured IAs who did not undergo radiological examination were not included. The frequency of drug use in these patients may be lower than that in patients diagnosed with unruptured IAs because these patients may not visit hospitals on a regular basis. Third, the association of several drug classes, such as angiotensin-converting enzyme inhibitors, peroxisome proliferator-activated receptor γ agonists, and selective estrogen receptor modulators, could not be assessed adequately with the present study design because drug use in the included subjects was infrequent. Finally, in the present study, statins, Ca channel blockers, and ARBs were significantly associated with rupture status. However, the mechanisms could not be assessed because the blood pressure and serum cholesterol values were not included in the evaluation. Hypertension is a well-established risk factor of aSAH [25], and dyslipidemia may also promote the pathogenesis of IAs [4, 26, 27]. The underlying mechanisms of statins may thus be attributable to either lipid-lowering or pleiotropic effects, such as anti-inflammatory or anti-oxidant actions [28]. Ca channel blockers and ARBs may suppress rupture through either lowering systemic blood pressure or suppressing biological processes in the disease microenvironment, as suggested by animal studies [29].

## Conclusions

The present cross-sectional study investigated candidate drugs to prevent IA rupture. Our data suggest that statins, Ca channel blockers, and ARBs are candidate drugs for preventive treatment of unruptured IAs. In addition, non-aspirin NSAIDs should be carefully used in patients with unruptured IAs because these drugs may promote rupture. Overall, our study provides valuable insights for establishing drug treatment to prevent IA rupture.

## Supporting information

S1 Table (XLSX)

S2 Table (XLSX)
